# Loading of Polydimethylsiloxane with a Human ApoB-Derived Antimicrobial Peptide to Prevent Bacterial Infections

**DOI:** 10.3390/ijms23095219

**Published:** 2022-05-07

**Authors:** Maria De Luca, Rosa Gaglione, Bartolomeo Della Ventura, Angela Cesaro, Rocco Di Girolamo, Raffaele Velotta, Angela Arciello

**Affiliations:** 1 Department of Chemical Sciences, University of Naples Federico II, 80126 Naples, Italy; maria.deluca2@unina.it (M.D.L.); rosa.gaglione@unina.it (R.G.); angela.cesaro@unina.it (A.C.); rocco.digirolamo@unina.it (R.D.G.); 2 Istituto Nazionale di Biostrutture e Biosistemi (INBB), 00136 Rome, Italy; 3 Department of Physics “E. Pancini”, University of Naples Federico II, 80126 Naples, Italy; bartolomeo.dellaventura@unina.it (B.D.V.); raffaele.velotta@unina.it (R.V.); 4 Machine Biology Group, Departments of Psychiatry and Microbiology, Institute for Biomedical Informatics, Institute for Translational Medicine and Therapeutics, Perelman School of Medicine, University of Pennsylvania, Philadelphia, PA 19104, USA; 5 Departments of Bioengineering and Chemical and Biomolecular Engineering, School of Engineering and Applied Science, University of Pennsylvania, Philadelphia, PA 19104, USA; 6 Penn Institute for Computational Science, University of Pennsylvania, Philadelphia, PA 19104, USA

**Keywords:** antimicrobial peptides, host defense peptides, polydimethylsiloxane, bacterial infections, biofilms, antimicrobial resistance, antimicrobial surfaces, infection prevention

## Abstract

Background: medical device-induced infections affect millions of lives worldwide and innovative preventive strategies are urgently required. Antimicrobial peptides (AMPs) appear as ideal candidates to efficiently functionalize medical devices surfaces and prevent bacterial infections. In this scenario, here, we produced antimicrobial polydimethylsiloxane (PDMS) by loading this polymer with an antimicrobial peptide identified in human apolipoprotein B, r(P)ApoB_L_^Pro^. Methods: once obtained loaded PDMS, its structure, anti-infective properties, ability to release the peptide, stability, and biocompatibility were evaluated by FTIR spectroscopy, water contact angle measurements, broth microdilution method, time-killing kinetic assays, quartz crystal microbalance analyses, MTT assays, and scanning electron microscopy analyses. Results: PDMS was loaded with r(P)ApoB_L_^Pro^ peptide which was found to be present not only in the bulk matrix of the polymer but also on its surface. ApoB-derived peptide was found to retain its antimicrobial properties once loaded into PDMS and the antimicrobial material was found to be stable upon storage at 4 °C for a prolonged time interval. A gradual and significant release (70% of the total amount) of the peptide from PDMS was also demonstrated upon 400 min incubation and the antimicrobial material was found to be endowed with anti-adhesive properties and with the ability to prevent biofilm attachment. Furthermore, PDMS loaded with r(P)ApoB_L_^Pro^ peptide was found not to affect the viability of eukaryotic cells. Conclusions: an easy procedure to functionalize PDMS with r(P)ApoB_L_^Pro^ peptide has been here developed and the obtained functionalized material has been found to be stable, antimicrobial, and biocompatible.

## 1. Introduction

### 1.1. Catheter-Associated Urinary Tract Infections

Infectious diseases related to the urinary tract represent the most frequent hospital-associated infections (HAIs) as reported by the World Health Organization (WHO) [1]. HAIs, despite being an important cause of morbidity and mortality, have a great financial impact due to prolonged patient hospitalization and treatment [2,3]. It has been reported that approximately 65–70% of the infections are observed in patients with urinary catheters [4], medical devices widely diffused in modern medical practice in the case of incontinence problems or post-operative urine retention [5]. Obviously, the risk of infection increases along with the period of permanence of the catheter inside the body [6]. The Center for Disease Control and Prevention defined catheter-associated urinary tract infections (CAUTIs) as the appearance of bacteria or fungi in the urine at concentrations higher than 105 CFUmL^−1^ [7]. The spread of the infection is mainly due to the formation of biofilm on the inner and outer surfaces of catheters, with the consequent invasion and colonization of the bladder by pathogens. Indeed, immediately after the insertion, the surfaces of catheters are altered by the deposition of urine components, such as proteins, minerals, and polysaccharides, a condition that favors the interaction and the adhesion of bacterial cells to catheters surfaces [8]. *Escherichia coli*, *Pseudomonas aeruginosa*, *Enterococcus faecalis*, *Proteus mirabilis*, and *Staphylococcus aureus* are the strains commonly recovered from infected catheters [9,10]. Microbial contamination and biofilm formation are the main reasons for the failure of indwelling medical devices since they cause devices’ encrustation and blockage [11]. An important issue is the difficulty to avoid bacterial colonization of medical devices surfaces despite rigorous hygiene procedures [12]. This is really worrying also because the treatment with conventional antibiotics is often ineffective to prevent and treat bacterial contaminations [13]. Since bacteria contaminating the bladder can also reach and infect other sites of the body, such as kidneys (pyelonephritis) and bloodstream (septicemia), this phenomenon might lead to sepsis and, in extreme cases, also to death [12].

### 1.2. Anti-Infective Biomaterials

Based on this, alternative strategies able to efficiently inhibit the adherence of pathogens to catheters surfaces are urgently needed. In this scenario, the use of anti-infective biomaterials able to either counteract the adhesion of microbes or kill them in the surrounding areas is gaining increasing attention. To date, several compounds, such as antibiotics [14], quaternary ammonium compounds [15], metals, and metal nanoparticles [16], have been employed to confer antimicrobial properties to the surfaces of medical devices. Among these, silver has been approved by the Food and Drug Administration (FDA) as a suitable antimicrobial tool to coat urinary catheters (i.e., silver-coated Dover™ catheters) [17]. Catheters loaded with the antimicrobial compound nitrofural are also commercially available with the name of Rochester Medical Magic 3 nitrofurazone-catheters [18]. However, it has to be highlighted that the employment of these functionalized materials is limited by their low efficiency, toxicity, and the development of bacterial resistance phenotype [19,20].

### 1.3. Antimicrobial Peptides

In this scenario, antimicrobial peptides (AMPs) are emerging as interesting effective tools to functionalize surfaces and fabricate materials with inherent antimicrobial properties. As a part of the innate immune system, AMPs exhibit a broad-spectrum range of activities and provide a first line of defense in virtually all organisms [21]. They generally do not lead to the selection of resistant bacterial strains [22], since their main target is the cell membrane and the acquisition of resistance would require an almost complete remodeling of bacterial membrane architecture [23]. In the present work, peptide r(P)ApoB_L_^Pro^, derived from an antimicrobial region encrypted in the sequence of human apolipoprotein B [24], has been selected to produce antimicrobial polydimethylsiloxane (PDMS). Indeed, by using a bioinformatics tool, we identified an antimicrobial region (amino acids 887–922) within the sequence of human ApoB [24]. Next, we produced recombinantly in *Escherichia coli* three versions of the identified encrypted peptide, namely r(P)ApoB_L_^Pro^, r(P)ApoB_S_^Pro^, and r(P)ApoB_L_^Ala^ [25]. These sequences present a Pro residue at the N-terminal extremity because of the acidic cleavage of an Asp-Pro bond necessary to excise peptides of interest [24]. The ApoB peptide variants were labeled with Pro and Ala indicating their amino acid residue in position 7 corresponding to the mutation that differentiates the two isoforms. The labels L and S refer to a longer or a shorter version of the identified amino acid sequence and correspond to the relative and absolute scores, respectively, generated by the used algorithm [24]. r(P)ApoB_L_^Pro^ peptide was here selected on the basis of its interesting bioactivities, including antimicrobial, anti-biofilm, antifungal, wound healing, and immunomodulatory properties [24,25,26,27,28,29,30,31,32,33]. Moreover, r(P)ApoB_L_^Pro^ peptide was found to be able to synergistically act in combination with conventional antibiotics and EDTA, demonstrating bactericidal activity in combination therapies at lower therapeutic doses and at shorter times of treatment in comparison with conditions in which it is administered as a single agent [24].

### 1.4. Functionalization of PDMS with an ApoB-Derived Antimicrobial Peptide

Based on these interesting properties of r(P)ApoB_L_^Pro^, the peptide was here selected to functionalize PDMS, a chemically stable, elastic, transparent, and biocompatible polymer representing one of the materials of choice for the production of biomedical devices, such as catheters and drainage tubes, dialysis membranes, micro pumps, micro valves, fluidic circuits and optical systems (adaptive lenses) [34]. Generally, two kinds of catheterizations are possible, i.e., (i) the “intermittent” catheterization in which the catheter is replaced by a novel one several times (range 1–10) per day and (ii) the “indwelling” catheterization, in which a balloon plays the key role to anchor the catheter in the bladder for weeks before its replacement [10]. In the latter case, they can be identified as (i) short-term catheters that are employed for temporary relief of reversible bladder voiding difficulties, for urine output monitoring or after urinary tract surgery, and are used in a range of time comprised between 1 and 14 days [17], or (ii) long-term catheters, used to manage chronic urinary retention or incontinence not treatable by different strategies, that might remain in place for up to three months [35]. In any case, catheters are exposed to human urine composed primarily of water (95%) containing urea (2%), creatinine (0.1%), uric acid (0.03%), sodium chloride, potassium, sulphate, ammonium, phosphate, and other ions and molecules in lesser amounts, with a pH value comprised in the range 4.5–8. Indeed, the composition of urine is not stable over time but varies throughout the same day and on the basis of age, gender, and race [36]. Urinary catheters employed in medicine are generally composed of polyvinyl chloride (PVC), polyurethanes, silicone, latex rubbers, and polydimethylsiloxane (PDMS) [37]. The composition of the PDMS employed in the present work was designed to be analogous to that of PDMS used to fabricate medical catheters. PDMS functionalized with r(P)ApoB_L_^Pro^ peptide was here produced and its anti-infective and biocompatibility properties were analyzed.

## 2. Results

### 2.1. Characterization of Functionalized PDMS by Fourier-Transform Infrared (FTIR) Spectroscopy

**Figure 1:**
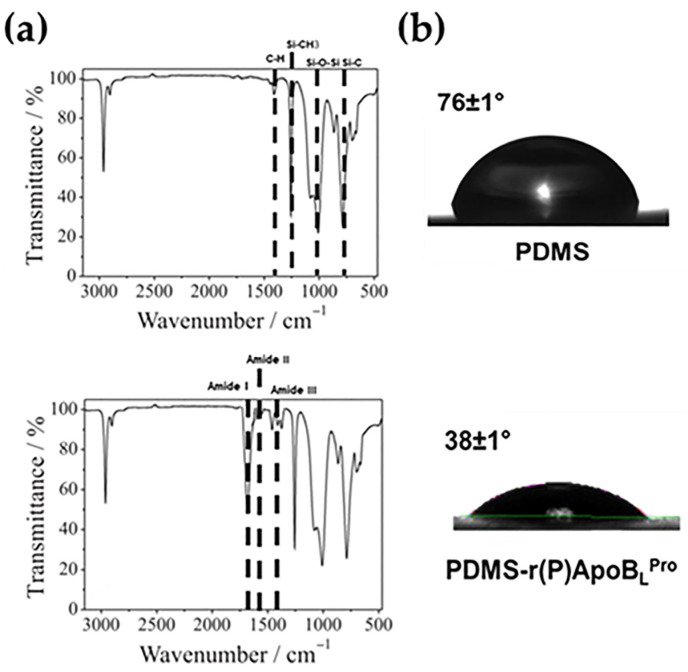
FTIR analyses of PDMS alone (upper spectrum) and of PDMS functionalized with r(P)ApoB_L_^Pro^ (lower spectrum) (**a**). Water contact angle analyses of PDMS alone (upper image) and of PDMS functionalized with r(P)ApoB_L_^Pro^ (lower image) (**b**).

FTIR spectroscopy was employed to analyze PDMS polymer upon functionalization with r(P)ApoB_L_^Pro^ peptide. To this purpose, FTIR spectra were collected in attenuated total reflection (ATR) mode. The spectra obtained for PDMS slides unfunctionalized or functionalized with r(P)ApoB_L_^Pro^ peptide are reported in Figure 1a. In the case of functionalized PDMS, obtained spectra were characterized by the contribution of amide I (at 1640 cm^−1^), amide II (at 1539 cm^−1^), and amide III (at about 1248 cm^−1^) (lower spectrum in Figure 1a), clearly indicating the presence of ApoB-derived peptide on PDMS surface. Indeed, these signals were found to be absent in the case of unfunctionalized PDMS (upper spectrum in Figure 1a). Indeed, in this case, the most intense bands of the spectrum are observed at 1052 cm^−1^ for asymmetric stretching of the Si–O–Si chain. This band is generally located between 1200 and 1000 cm^−1^ and has a typical asymmetric shape ascribed to the presence of silica. The broad shoulder observed around 1157 cm^−1^ can be related to vibrations of cyclic species as widely reported [38]. The symmetric stretching of the Si–O–Si chain appears at 79 cm^−1^, whereas the band at 954 cm^−1^ is usually attributed to the Si–O stretch of silanols. It has to be highlighted that, to verify the homogeneity of analyzed samples, ten spectra have been collected for cross-sectioned sample surfaces. All the obtained spectra were found to be very similar, thus indicating the homogeneity of analyzed unfunctionalized samples.

### 2.2. Water Contact Angle Analyses of Functionalized PDMS

To verify whether modifications in surface wettability occur upon PDMS functionalization with r(P)ApoB_L_^Pro^ peptide, water contact angle measurements were performed. A surface can be defined as “hydrophilic” when the water contact angle (WCA) is lower than 90°, whereas it is considered “hydrophobic” when WCA is comprised between 90° and 180°. PDMS alone is known to be naturally hydrophobic although, here, it was found to be slightly hydrophilic (WCA = 76 ± 1°) due to the plasma activation procedure performed during PDMS preparation (Figure 1b, upper image). Upon PDMS functionalization with r(P)ApoB_L_^Pro^ peptide, interestingly, the surface hydrophilicity was found to increase as indicated by the decrease in the obtained value (WCA = 38 ± 1°, Figure 1b, lower image). This is a clear indication of the effective presence of ApoB-derived peptide on the surface of PDMS upon functionalization procedure.

### 2.3. Evaluation of the Antimicrobial Activity of PDMS Functionalized with r(P)ApoB_L_^Pro^

**Figure 2:**
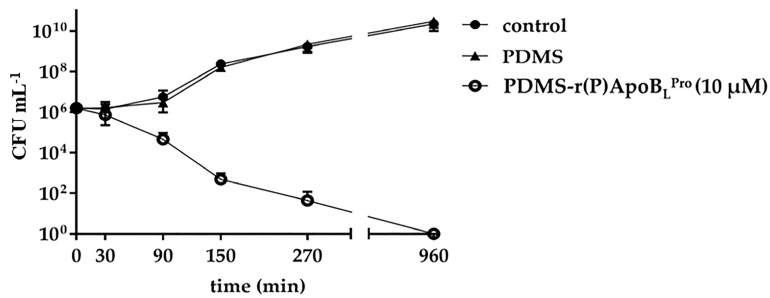
Time killing curves obtained by incubating *E. coli* ATCC 25922 cells alone (control) or in the presence of unfunctionalized PDMS or with PDMS-r(P)ApoB_L_^Pro^ (10 µM). Data represent the mean (±SD) of at least two independent experiments, each one carried out with triplicate determinations.

In order to evaluate the antimicrobial performance of PDMS functionalized with the selected ApoB-derived peptide, minimal bactericidal concentration (MBC) values were determined for functionalized PDMS in comparison with those obtained for peptide alone tested against *Escherichia coli* ATCC 25922 and *Staphylococcus aureus* ATCC 29213 bacterial strains (Table 1), selected as representatives of Gram-negative and Gram-positive bacteria, respectively. As shown in Table 1, PDMS functionalized with r(P)ApoB_L_^Pro^ peptide, here named PDMS-r(P)ApoB_L_^Pro^, was found to exert antimicrobial effects even if higher concentrations of peptide were required to completely kill bacterial cells with respect to peptide tested alone in solution (Table 1). It is noteworthy that PDMS-r(P)ApoB_L_^Pro^ was found to be more active against the Gram-negative *E. coli* ATCC 25922 strain, which was selected as a model strain for further investigations. In order to analyze the kinetic of the bactericidal activity of functionalized PDMS, kinetic killing assays were also performed. To this purpose, at selected time intervals (i.e., 0–30–90–150–270–960 min), the growth of *E. coli* ATCC 25922 cells cultivated in contact with PDMS alone or with PDMS-r(P)ApoB_L_^Pro^ was evaluated by colonies counting. As shown in Figure 2, a progressive decrease in bacterial cells viability was detected only in the presence of PDMS-r(P)ApoB_L_^Pro^ (10 µM). The strongest effects on bacterial growth were observed after 270 min of incubation, with a complete death of bacterial cells after 960 min of incubation with PDMS-r(P)ApoB_L_^Pro^ (10 µM).

### 2.4. Evaluation of Peptide Release by Quartz Crystal Microbalance (QCM) Analyses

**Figure 3:**
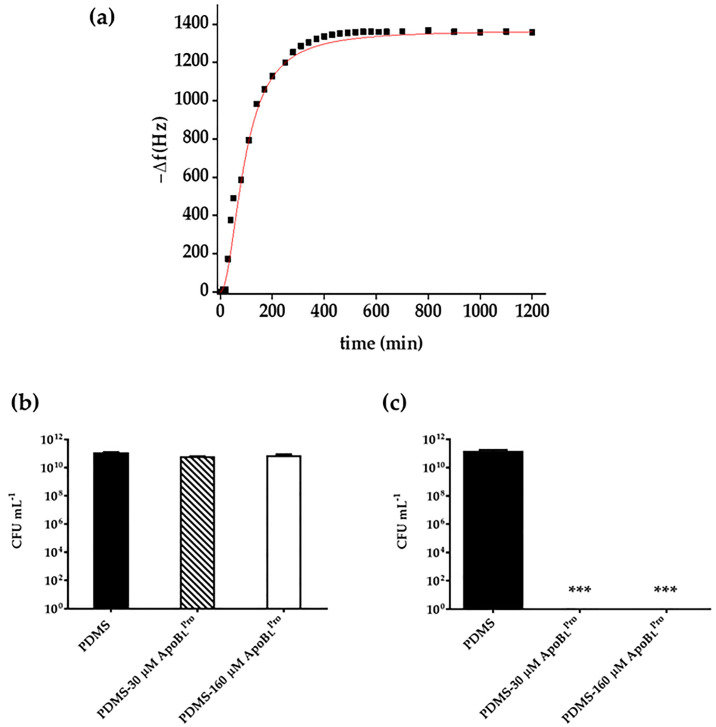
Kinetic analyses of r(P)ApoB_L_^Pro^ release from PDMS (**a**). The obtained red curve is the best fit of the experimental values (black squares) obtained by a logistic function. Evaluation of the antibacterial activity of PDMS functionalized with two different concentrations (30 and 160 μM) of r(P)ApoB_L_^Pro^ peptide and incubated with 0.5X nutrient broth (NB) for 400 min at 37 °C. Upon incubation, both PDMS (**b**) and the medium containing released peptide (**c**) were tested for their activity towards *E. coli* ATCC 25922. Data represent the mean (±SD) of at least two independent experiments, each one carried out with triplicate determinations. Significant differences were indicated as *** (*p* < 0.001).

In order to verify whether r(P)ApoB_L_^Pro^ peptide is released from the PDMS surface upon functionalization, QCM analyses were performed. The experiments were conducted by directly depositing functionalized PDMS on the quarts. In these experimental conditions, the first recorded value corresponds to the resonant frequency of the electrode. Afterwards, 300 µL of pure water were added to the surface and removed after a fixed time, in order to record the newly reached frequency value and to obtain data on peptide release. This analysis was repeated at defined time intervals, in order to collect data indicative of the release of the peptide from the PDMS surface into the medium. As shown in Figure 3a, a fast release was observed, since about 70% of the peptide initially used to functionalize PDMS was released after 400 min. This could be due to the hydrophobic properties of the PDMS polymer that is unable to establish electrostatic interactions with cationic peptide molecules. As a consequence of this, the PDMS polymer might act as a reservoir for the peptide. Considering the molecular weight of the peptide (4075 Da) and the relationship to compare the frequency variation with bound mass (Kanazawa’s equation) on the transducer (quartz) that corresponds to 1 Hz at about 1.1 ng, we are able to affirm that about 70% release of the peptide (1.4 µg) on the initial mass (2 µg) was obtained. To evaluate whether functionalized PDMS retains its antimicrobial properties upon peptide release, PDMS was functionalized by using two different concentrations (30 and 160 μM) of r(P)ApoB_L_^Pro^ peptide and incubated with 0.5X nutrient broth (NB) for 400 min at 37 °C. Upon incubation, both PDMS and the medium containing released peptide were tested for their activity towards the *E. coli* ATCC 25922 bacterial strain (Figure 3b,c). As expected, no antimicrobial activity was detected in the case of PDMS upon peptide release (Figure 3b), whereas a complete cell death was obtained when bacterial cells were incubated with the medium containing all the peptide released during 400 min incubation (Figure 3c), thus indicating the efficacy of the here developed system in preventing bacterial infections during catheters implantation.

### 2.5. Storage Stability of PDMS Functionalized with r(P)ApoB_L_^Pro^ Peptide

**Figure 4:**
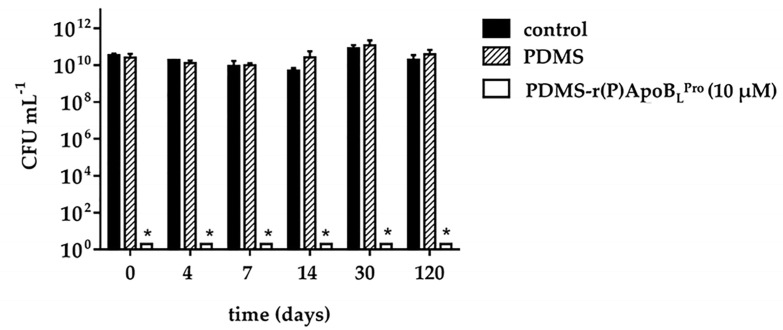
Evaluation of the ability of PDMS-r(P)ApoB_L_^Pro^ to retain its antimicrobial efficacy over time. Data represent the mean (±SD) of at least three independent experiments, each one carried out with triplicate determinations. Significant differences were indicated as * (*p* < 0.05).

In order to evaluate whether functionalized PDMS retains its antimicrobial properties over time, samples of PDMS functionalized with 10 µM r(P)ApoB_L_^Pro^ were prepared and stored at 4 °C. Following incubation for defined time lengths (4–7–14–30–120 days), the efficacy of samples to counteract bacterial growth was evaluated. Interestingly, as reported in Figure 4, PDMS-r(P)ApoB_L_^Pro^ was found to completely retain its antimicrobial efficacy after 120 days of storage at 4 °C, thus indicating that obtained functionalized material is endowed with great stability.

### 2.6. Anti-Infective Properties of PDMS Functionalized with r(P)ApoB_L_^Pro^ Peptide

**Figure 5:**
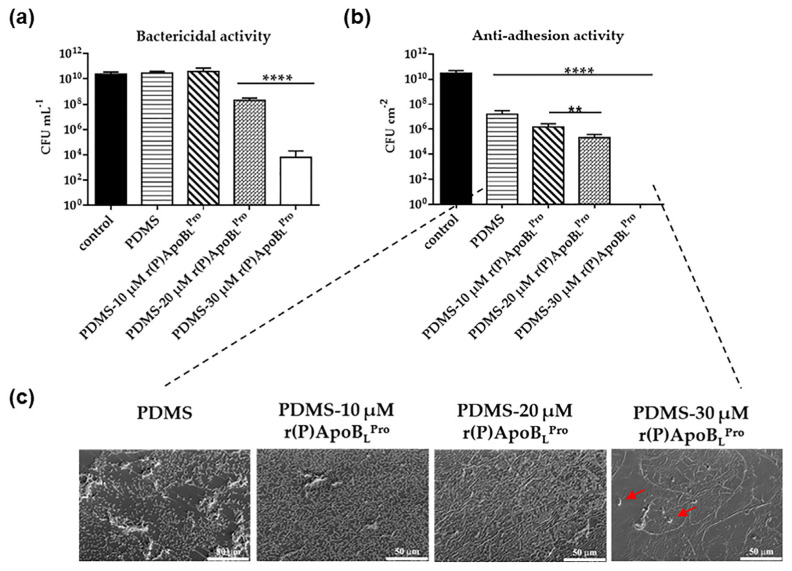
Analysis of the bactericidal activity of PDMS-r(P)ApoB_L_^Pro^ (**a**). Evaluation of PDMS-r(P)ApoB_L_^Pro^ ability to interfere with bacterial cells adhesion (**b**). SEM analyses of unfunctionalized PDMS and of PDMS-r(P)ApoB_L_^Pro^ upon incubation with *E. coli* ATCC 25922 bacterial cells (**c**). Cells debris (red arrows) is visible. Statistical analyses were performed by using Student’s *t*-test. Significant differences were indicated as ** (*p* < 0.01) or **** (*p* < 0.0001).

Bacterial adhesion is the first step of biofilm formation and colonization of surfaces [12]. To evaluate the ability of PDMS-r(P)ApoB_L_^Pro^ to prevent and interfere with bacteria adhesion, PDMS slides of 1 cm^2^ were mixed with increasing concentrations of r(P)ApoB_L_^Pro^ (10–20–30 µM) and incubated with *E. coli* ATCC 25922 bacterial cells. Following 16 h of incubation, colonies counting analyses were performed. Strong anti-infective properties were detected in the case of PDMS-r(P)ApoB_L_^Pro^ characterized by the highest peptide concentration tested (30 µM) (Figure 5). Indeed, under the experimental conditions tested, both bactericidal effects (Figure 5a) and the ability to counteract bacteria adhesion (Figure 5b) were detected. Bactericidal activity and anti-adhesive properties were evaluated by counting bacterial colonies deriving from bacterial cells remaining in the supernatant upon contact with the surface or from bacterial cells detached from the surface, respectively. Experimental details are described in the Section 4. It has to be highlighted that, although PDMS alone has no effects on the viability of bacterial cells, it is endowed with slight anti-adhesive properties probably because of plasma activation performed during material preparation (Figure 5b). Scanning electron microscopy (SEM) analyses were also carried out in order to further confirm the anti-adhesive properties of PDMS-r(P)ApoB_L_^Pro^. It was observed that, in the case of PDMS slides functionalized with 30 µM r(P)ApoB_L_^Pro^, no viable bacterial cells were detected, being present only bacterial cell debris (red arrows in Figure 5c) on the surface of the material. In the case of PDMS alone, viable bacterial cells are clearly evident, thus indicating that the presence of the peptide is responsible for cell death and for the inhibition of bacterial cells adhesion.

### 2.7. Anti-Biofilm Activity of PDMS Functionalized with r(P)ApoB_L_^Pro^ Peptide

**Figure 6:**
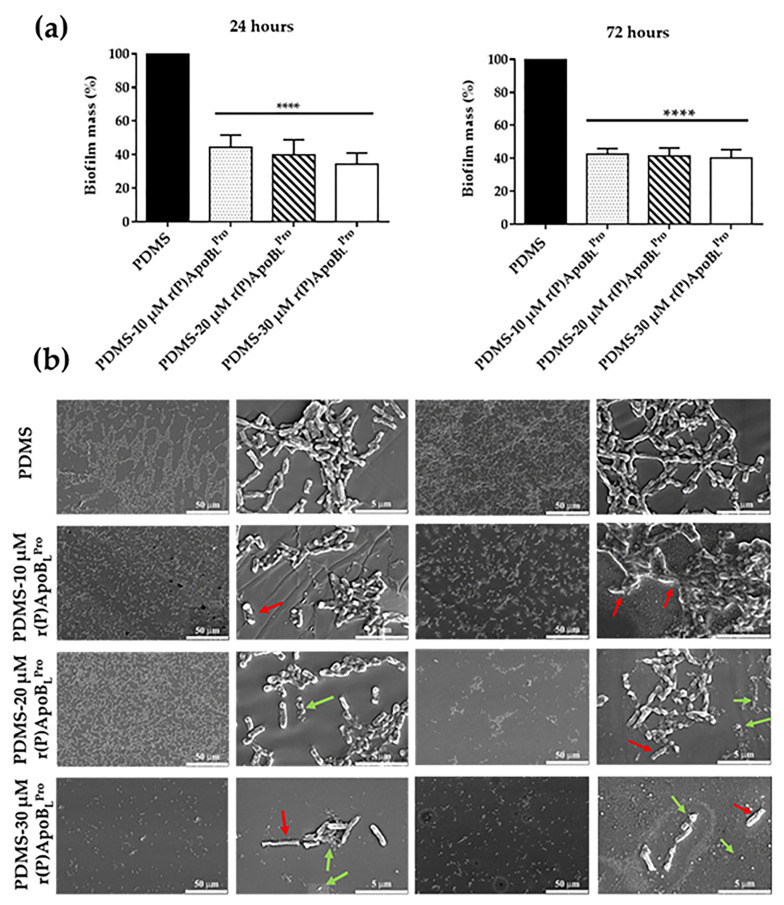
Evaluation of PDMS-r(P)ApoB_L_^Pro^ anti-biofilm activity by crystal violet assay (**a**) and SEM analyses (**b**). Red arrows indicate altered bacterial cells present in the case of functionalized PDMS; cell debris are indicated by green arrows. Statistical analyses were performed by using Student’s *t*-test. Significant differences were indicated as **** (*p* < 0.0001).

Anti-infective properties of functionalized PDMS were evaluated upon incubation with bacterial suspensions characterized by high cell density, since it has been reported that, in the case of urogenital infections, the bacterial load may be very high (up to 1 × 10^8^ CFU mL^−1^) [39]. For this reason, PDMS functionalized with r(P)ApoB_L_^Pro^ was incubated with *E. coli* ATCC 25922 (1 × 10^9^ CFU mL^−1^), in order to create conditions that favor biofilm mass formation on the PDMS surface. After 24 or 72 h incubation, crystal violet assays (CV) and scanning electron microscopy (SEM) analyses were performed. Crystal violet assays indicated a significant decrease in bacterial biomass accumulated on the surface of functionalized PDMS with respect to unfunctionalized PDMS (Figure 6a). Experiments were conducted by functionalizing the PDMS surface with increasing concentrations of ApoB-derived peptide (10–20–30 µM) and by incubating surfaces with bacterial cells for 24 or 72 h. Obtained results clearly indicate that the presence of the peptide on the PDMS surface is responsible for the significant inhibition of biofilm attachment under the experimental conditions tested (Figure 6a). To deepen this aspect, SEM analyses were also performed by using identical experimental conditions. As shown in Figure 6b, the density of bacterial cells significantly decreases in the presence of the peptide with the strongest effects observed at the highest peptide concentration tested (30 µM). It has also to be highlighted that, in the case of functionalized surface, bacterial cells appear translucent and characterized by corrugated and wrinkled surfaces (red arrows in Figure 6b) and not turgid and with regular and smooth surfaces as in the case of unfunctionalized PDMS (Figure 6b). Furthermore, widespread cell debris and cell fragments are also evident in the case of functionalized PDMS (green arrows in Figure 6b). It is also noteworthy that cells on PDMS alone appear encased in a matrix, indicative of the presence of biofilm. The absence of this matrix in the case of functionalized PDMS supports the hypothesis that ApoB-derived peptide present on the surface of PDMS is responsible for a significant inhibition of biofilm attachment.

### 2.8. Biocompatibility of PDMS Functionalized with r(P)ApoB_L_^Pro^ Peptide

**Figure 7:**
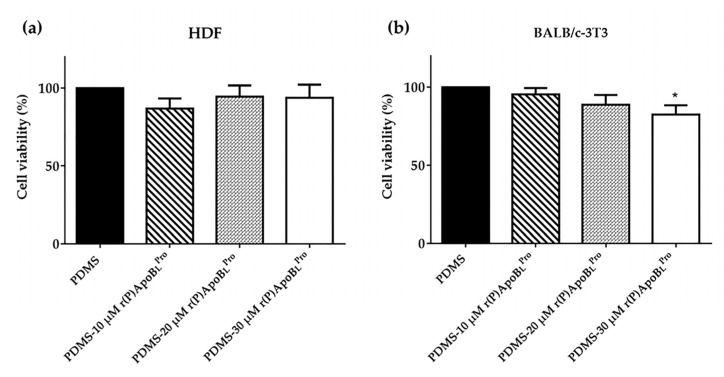
Analysis of the effects of PDMS loaded with increasing concentrations of r(P)ApoB_L_^Pro^ on the viability of human dermal fibroblasts (HDF) (**a**) and of murine BALB/c-3T3 fibroblasts (**b**). Cell viability was assessed by MTT assays and expressed as the percentage of viable cells with respect to cells incubated with unfunctionalized PDMS. Experiments were performed three times. Error bars represent the standard deviation of the mean. Significant differences were indicated as * (*p* < 0.05) for treated *vs.* control samples.

Being medical devices in constant contact with human body tissues, they have to satisfy safety requisites. For this reason, the cytocompatibility of functionalized PDMS was evaluated *in vitro* towards murine BALB/c-3T3 fibroblasts and human dermal fibroblasts (HDFs) by MTT assays. To this purpose, PDMS slides functionalized with increasing concentrations of r(P)ApoB_L_^Pro^ (10–20–30 µM) were incubated with selected cells for 24 h. Interestingly, functionalized PDMS samples were found not to affect the viability of tested eukaryotic cells under the experimental conditions tested (Figure 7a,b) and slight toxic effects (about 20% cell death) were detected only on murine BALB/c-3T3 fibroblasts at the highest peptide concentration tested (30 µM) (Figure 7b).

## 3. Discussion

Catheters are medical devices routinely utilized in modern health care. However, their use is commonly associated with infections, known as catheter-associated urinary tract infections (CAUTIs), causing a significant burden on healthcare [40]. This is alarming also considering that treatments with conventional antibiotics are often ineffective due to the spread of resistance phenotypes [41] and that infections associated with biofilm formation are more difficult to manage since bacteria embedded into biofilms are up to 1000 times more resistant to antimicrobial therapy with respect to their planktonic counterparts [42]. To reduce CAUTIs caused by bacterial adhesion on catheters surfaces, the use of materials endowed with antimicrobial properties has attracted great attention [19,43]. Although catheters coated with antimicrobial compounds are currently commercially available, their efficacy in clinics is far from satisfying. Indeed, they must face important problems, such as high costs of synthesis and putative toxicity [44]. For this reason, there is an urgent need for alternative and effective strategies to avoid medical devices infection and failure. In this scenario, the use of AMPs to create anti-infective materials appears promising because of the peculiar properties of these peptides [45]. An important issue is also the low probability of resistance development upon a prolonged exposure of bacterial cells to AMPs [46]. Polydimethylsiloxane (PDMS) elastomer is a material widely used for biomedical applications because of its excellent biocompatibility and mechanical properties [34]. However, PDMS-based biomedical devices are susceptible to microbial adhesion and invasion [47]. Here, a simple method has been employed to functionalize PDMS with an ApoB-derived peptide and the anti-infective properties of the obtained material have been investigated. First of all, once obtained PDMS loaded with ApoB-derived peptide [PDMS-r(P)ApoB_L_^Pro^], its structural features have been investigated by FTIR spectroscopy and water contact angle analyses, which confirmed the presence of the peptide not only in the bulk matrix of the polymer, as supposed according to the functionalization procedure, but also on the surface of the material. It has to also be highlighted that the altered water contact angle, indicative of an increase in surface hydrophilicity upon the incorporation of the peptide into PDMS, surely alters the properties of the catheter surface by providing an advantage over conventional PDMS catheters. Indeed, it has been widely reported that catheters insertion, especially in the case of intermittent catheterization, is associated with pain for the patient. To avoid this, catheters are generally lubricated to reduce the friction on the urethral wall and to prevent trauma occurrence and pain. To reach this goal, glycerine, lidocaine gels, or hydrophilic coatings are generally applied on catheter surfaces to increase its hydrophilicity [48]. For this reason, the increased surface hydrophilicity registered upon the incorporation of the peptide into PDMS might be considered an improvement over PDMS conventional material, since it might promote a smooth catheterization and might allow avoiding external lubrication that can cause catheter bacterial contamination prior to its insertion [10]. In the present work, antimicrobial assays have also been performed to evaluate whether the functionalized material is endowed with antimicrobial activity. To this purpose, a Gram-positive and a Gram-negative bacterial strain, i.e., *E. coli* ATCC 25922 and *S. aureus* ATCC 29213, have been selected. Interestingly, functionalization of PDMS with r(P)ApoB_L_^Pro^ peptide confers to the material antimicrobial properties, with the strongest effects observed in the case of the Gram-negative *E. coli* ATCC 25922 bacterial strain. Indeed, in the case of the Gram-positive *S. aureus* ATCC 29213 bacterial strain, PDMS-r(P)ApoB_L_^Pro^ was found to be effective at concentrations significantly higher than the MIC value determined for the peptide in solution. For this reason, the antimicrobial properties of the functionalized PDMS were further analyzed by using the most promising bacterial strain, i.e., *E. coli* ATCC 25922. It has been demonstrated that the functionalized material retains its antimicrobial properties if stored at 4 °C for prolonged time intervals, thus opening interesting perspectives to the applicability of this material in the biomedical field. The release of the peptide from PDMS was also evaluated over time by QCM analyses indicating a gradual and significant release of the peptide (70% of the total amount) after an incubation of 400 min. This observation opens interesting perspectives to the future applicability of the peptide that, once released from the surface of an implanted medical device, might reach the surrounding tissue areas, thus preserving them from infections. However, it has to be highlighted that PDMS losses its antimicrobial properties upon peptide release. Indeed, experiments were performed by functionalizing PDMS with two different concentrations (30 and 160 μM) of r(P)ApoB_L_^Pro^ peptide and incubating it with 0.5X nutrient broth (NB) for 400 min at 37 °C. Upon incubation, both PDMS and the medium containing the released peptide were tested for their activity towards *E. coli* ATCC 25922 (Figure 3b,c). No antimicrobial activity was detected in the case of PDMS upon peptide release, whereas a complete cell death was obtained when bacterial cells were incubated with the medium containing all the peptide released during 400 min incubation, thus indicating that the peptide released in the surrounding medium is highly able to counteract bacterial infections. Hence, the here developed system appears effective in preventing bacterial infections during catheters implantation and in the case of short-term catheters. Bactericidal and anti-infective properties of functionalized PDMS have been also evaluated by preparing slides (1 cm^2^ surface area and 0.3 cm thickness) of functionalized PDMS that were analyzed and found to be endowed with strong anti-adhesive properties being able to prevent the adhesion of bacterial cells and exerting strong bactericidal effects. Scanning electron microscopy (SEM) analyses have been also performed and confirmed the anti-adhesive properties of functionalized PDMS. Altogether, these findings suggest the ability of the peptide, once enclosed into a material, to prevent bacteria colonization, a phenomenon often cited as the most common reason for medical devices failure [11]. Since the adhesion of bacterial cells to a surface is the first step in biofilm formation, the anti-adhesive properties of this novel functionalized material have been also deepened by performing, as first, crystal violet assays indicating the ability of functionalized PDMS to counteract the adhesion of bacterial cells. This was also confirmed by SEM analyses that evidenced a strong anti-adhesive activity exerted by the highest peptide concentration tested, thus suggesting that the observed inhibition of biofilm attachment could be related to the ability of the peptide to directly kill bacterial cells. Indeed, the peptide was found to be released upon PDMS functionalization and, once free in the surrounding environment, could act by directly killing bacteria, thus preventing their adhesion to the surface. The biocompatibility of obtained functionalized PDMS was also evaluated by testing its effects on the viability of eukaryotic cell lines. This is an important issue, considering that the introduction of an external device in the human body requires its full safety for human tissues [49]. *In vitro* assays indicated that PDMS functionalized with increasing concentrations of r(P)ApoB_L_^Pro^ peptide did not affect the viability of human dermal fibroblasts (HDF) and of murine BALB/c-3T3 fibroblasts, thus adding a further important tassel in favor of the applicability of this novel functionalized material. Altogether, obtained data demonstrate the applicability of ApoB-derived peptide to functionalized PDMS while preserving its antimicrobial properties. Several studies have been previously reported on the functionalization of surfaces of medical devices with AMPs. These studies are mainly based on the covalent immobilization of peptides. As an example, an N-terminus cysteine-modified Lasio-III AMP has been immobilized on the surface of a commercially available silicone Foley catheter by using a PEG spacer coupled to an AGE brush [50]. Two synthetic salt-resistant AMPs (RK1 and RK2) have been also successfully immobilized on PDMS surfaces via an allyl glycidyl ether (AGE) polymer brush [51]. Another AMP candidate, CWR11, has been immobilized on the surface of PDMS previously modified by coating with a polydopamine layer allowing the attachment of the peptide [52]. The synthetic cysteine-labeled peptide E6 (RRWRIVVIRVRRC) has been also immobilized on the surface of polyurethane catheters via a polymer brush coating [53]. In all the cases, the antimicrobial activity of the immobilized peptide molecules has been proved, and it has been demonstrated that several key factors strongly influence the antimicrobial performance of immobilized AMPs and should be considered for successful immobilization and peptide activity. These factors range from peptide coverage or surface density to orientation, peptide distance from the surface, or even peptide lateral mobility [17]. Although covalent-based approaches appear more advantageous and efficient in terms of antimicrobial activity, it has to be highlighted that the physical adsorption of AMPs on the surface of interest might ensure an active gradual release of the antimicrobial agent, a feature that might be preferred for some kinds of applications. Here, we present a proof-of-concept study based on the non-covalent incorporation of a natural ApoB-derived peptide into PDMS. Due to peptide fast release, the here developed system appears suitable to be employed in the case of short-term catheters, generally employed for temporary relief of reversible bladder voiding difficulties, for urine output monitoring, or after urinary tract surgery. These short-term catheters are used in a range of time comprised between 1 and 14 days and are generally not subjected to encrustation, due to the deposition on the catheter surface of the salts present in urine [17]. It has also to be highlighted that the method here developed to functionalize the PDMS polymer with ApoB-derived peptide is, in principle, suitable and applicable to any peptide molecule, thus opening interesting perspectives to the investigation of the applicability of different antimicrobial peptides to functionalize materials of medical interest.

## 4. Materials and Methods

### 4.1. Materials

Unless specified otherwise, all reagents used in the present study were purchased from Sigma-Merck (Milan, Italy).

### 4.2. Bacterial Strains and Growth Conditions

Bacterial strains *E. coli* ATCC 25922 and *S. aureus* ATCC 29213 were grown in Mueller–Hinton broth (MHB; Becton Dickinson Difco, Franklin Lakes, NJ, USA) and on tryptic soy agar (TSA; Oxoid Ltd., Hampshire, UK). In all the experiments, bacteria were inoculated and grown overnight in MHB at 37 °C. The next day, bacteria were transferred to a fresh MHB tube and grown to mid-logarithmic phase [24,30].

### 4.3. Peptide Production

Expression and isolation of the recombinant peptide r(P)ApoB_L_^Pro^ was carried out as previously described [25]. Briefly, *E. coli* BL21(DE3) cells were transformed with a pET recombinant plasmid and grown in 10 mL of terrific broth (TB) medium containing 100 µg/mL of ampicillin, at 37 °C up to an OD_600nm_ of 2. These cultures were used to inoculate 1 L of TB/ampicillin medium. Glucose at a final concentration of 4 g/L was added to cultures to limit protein expression before induction with IPTG. Cultures were incubated at 37 °C up to OD_600nm_ of 3.5–4. Expression of the recombinant protein was induced by the addition of IPTG (isopropyl-b-D-thiogalactopyranoside) at a final concentration of 0.7 mM. Cells were harvested after overnight induction by centrifugation at 8000× *g* for 15 min at 4 °C and washed with 50 mM Tris-HCl buffer pH 7.4. The bacterial pellet was suspended in 50 mM Tris-HCl buffer pH 7.4 containing 10 mM ethylenediaminetetraacetic acid (EDTA) and sonicated in a cell disruptor (10 × 1 min cycle, on ice). The suspension was then centrifuged at 18,000× *g* for 60 min at 4 °C. The insoluble fractions were washed three times in 0.1 M Tris-HCl buffer pH 7.4 containing 10 mM EDTA, 2% Triton X-100, and 2 M urea, followed by repeated washes in 0.1 M Tris-HCl buffer pH 7.4. Following washing steps, 100 mg of fusion protein was dissolved in 10 mL of denaturing buffer (6 M guanidine/HCl in 50 mM Tris-HCl, pH 7.4) containing 10 mM β-mercaptoethanol. The mixture was incubated at 37 °C for 3 h under nitrogen atmosphere on a rotary shaker and then centrifuged at 18,000× *g* for 60 min at 4 °C. Soluble fractions were then collected and purified by affinity chromatography on Ni Sepharose^TM^ 6 Fast Flow resin (GE Healthcare Lifescience, Chicago, IL, USA). The chromatographic fractions were analyzed by 15% SDS/PAGE, pooled, and extensively dialyzed against 0.1 M acetic acid pH 3 at 4 °C. Any insoluble material was removed by centrifugation and filtration. The sample containing the fusion construct was then acidified to pH 2.0 by the addition of 0.6 M HCl to allow the cleavage of the Asp-Pro linker peptide, purged with N_2_, and incubated at 60 °C for 24 h in a water bath. The pH was then increased to 7–7.2 by the addition of 1 M NH_3_ and incubated overnight at 28 °C to selectively precipitate the carrier ONC-DCless-H6, which is insoluble at neutral or alkaline pH values. The peptide was isolated from insoluble components by repeated cycles of centrifugation. A final gel-filtration step was added in order to remove salts used along the purification process that tend to attach to the peptide molecules. Following this step, the peptide was lyophilized and its purity was checked by SDS/PAGE and mass spectrometry analyses. Lyophilized peptide was dissolved in pure water, unless differently specified, and quantified by BCA assay (Thermo Fisher Scientific, Waltham, MA, USA).

### 4.4. Preparation of PDMS Polymer Loaded with r(P)ApoB_L_^Pro^ Peptide

To prepare PDMS polymer and functionalize it with r(P)ApoB_L_^Pro^ peptide, we followed four main steps as here described. (1) Pre-Treatment. Firstly, the silane groups of the silicone elastomer (Sylgard™ 184 silicone elastomer) were activated by using a low-frequency air plasma treatment for 1 min. The plasma-activated sample was rinsed by washing with ethanol for at least 10 min and this procedure was repeated 3 times. Then, it was air-dried for at least 15 min. (2) First Modification Step. The surface of the polymer was then covered with glycidyl methacrylate-based polymer, typically ~1 mL of the solution per square inch of the surface. The sample was then dried for 20–30 min at room temperature and annealed at 110 °C under nitrogen or argon for 30 min. The annealed sample was then rinsed three times with methylethylketone for 10 min each time. Afterwards, it was air-dried for at least 30 min. (3) Polymer Grafting. The polymer sample of step 2 was treated with a solution obtained by carefully stirring ethanol (6 mL) with acrylamide/acrylic acid-based polymer. Once the homogeneous solution was obtained, the surface of the horizontally positioned polymer was completely covered with it (~1 mL per square inch of the surface). (4) Polymer–peptide mixture preparation. The antimicrobial peptide was then added to the liquid polymeric solution obtained in the previous step (1:1 *v*/*v*) and stirred for 1 h at room temperature. After that, the obtained homogeneous polymeric solution was poured into multi-well plates and heated at 37 °C for 4–8 h to allow its solidification. Indeed, the functionalized PDMS was used in the cured form. All the performed steps, including the use of different polymers, are crucial to obtain the formation of a PDMS polymer network.

### 4.5. Characterization of PDMS Polymer Loaded with r(P)ApoB_L_^Pro^ Peptide by Fourier-Transform InfraTed (FTIR) Spectroscopy Analyses

Micro-ATR spectra were recorded by using a Perkin Elmer Spectrum One FTIR spectrometer equipped with a Perkin-Elmer Multiscope system infrared microscope (Mercury Cadmium Telluride detector). A 0.6 mm radius germanium hemispherical internal reflection element (IRE) was employed. The sampling area at the tip of the IRE coincided with the focal point of the infrared objective [50]. Spectra were acquired in the reflection mode, thus allowing to collect the signal coming from a few μm-thick sample layers. Under mechanical control, the miniature-Ge IRE was removed, in order to allow cleaning, and reinserted in the same pre-aligned position. In order to collect ATR FT-IR spectra from a flat surface with the miniature-Ge IRE, the samples were placed on the microscope stage equipped with a movable 75 mm × 50 mm XY stage. A specific spot on the surface was selected through a 10X optical or 15X infrared objective. The miniature-Ge IRE was then slid into the pre-aligned position and the microscope stage was elevated until the selected sampling area touched the tip of the IRE. Particular care was employed to obtain the same degree of contact for all the measurements. Once a good contact was obtained, ATR FT-IR spectra of the selected area were collected. Background spectra were collected through the IRE when it was not in contact with the sample. All the spectra were collected by using 16 scans in the range from 4000 to 600 cm^−1^ with a 4 cm^−1^ spectral resolution and a 5 s acquisition time for each spectrum. Ten spectra were collected for each sample. Measurements were carried out on PDMS layers (0.3 cm thick) in the presence or in the absence of the peptide as previously described [54]. Obtained spectra were preliminarily analyzed by using a proper software package (“Spectrum” User Guide, Perkin Elmer Inc., Waltham, MA, USA). The presence of the peptide was verified by plotting the areas of the bands corresponding to amide I and amide II (1580–1700 cm^−1^). A curve fitting of the amide I band into its respective secondary structure components was then performed. In particular, the band was analyzed in terms of the sum of six Lorentzian curves, whose characteristics (intensity, position, and width) were obtained by a nonlinear fitting minimization procedure following the Levenberg–Marquardt algorithm [55].

### 4.6. Characterization of PDMS Polymer Loaded with r(P)ApoB_L_^Pro^ Peptide by Water Contact Angle Measurements

Static water contact angle measurements were performed on unfunctionalized PDMS and on PDMS loaded with r(P)ApoB_L_^Pro^ peptide by a sessile drop method. A droplet (10 μL) of distilled water was pipetted onto each sample at room temperature. Contact angles were then measured by using a Theta Flex Optical Tensiometer and a digital protractor, which photographs the liquid interface and automatically calculates the angle. Three different surface spots were tested for each sample and the average of obtained values was reported.

### 4.7. Antimicrobial Activity Assays

The antimicrobial activity of r(P)ApoB_L_^Pro^ peptide and of PDMS functionalized with the peptide was tested towards two bacterial strains, i.e., *E. coli* ATCC 25922 and *S. aureus* ATCC 29213 by using the broth microdilution method [56]. In each case, bacteria were grown to mid-logarithmic phase in Mueller–Hinton broth (MHB; Difco, Becton Dickinson, Franklin Lakes, NJ, USA) at 37 °C and then diluted to 4 × 10^6^ CFUmL^−1^ in 0.5X Nutrient Broth (NB; Difco, Becton Dickinson, Franklin Lakes, NJ, USA). Afterwards, bacterial cells were mixed either with 1:1 *v*/*v* two-fold serial dilutions of the peptide (0–20 μM) or with PDMS functionalized with increasing concentrations of r(P)ApoB_L_^Pro^ peptide (0–80 μM). Following an overnight incubation, each sample was diluted, plated on tryptic soy agar (TSA), and incubated at 37 °C for 24 h, in order to count the number of colonies. By using this experimental procedure, MIC (minimal inhibitory concentration) values were determined as the lowest peptide concentration responsible for no visible bacterial growth, whereas MBC (minimal bactericidal concentration) values were determined as the lowest peptide concentration responsible for >99.9% cell death. To evaluate the antimicrobial activity of functionalized PDMS upon r(P)ApoB_L_^Pro^ peptide release, PDMS was functionalized by using two different concentrations (30 and 160 μM) of r(P)ApoB_L_^Pro^ peptide and incubated with 0.5X NB for 400 min at 37 °C. Upon incubation, both PDMS and the medium containing released peptide were tested for their activity towards the *E. coli* ATCC 25922 bacterial strain. In both cases, unfunctionalized PDMS was tested as a control. To perform the analysis, bacteria were grown to mid-logarithmic phase in MHB at 37 °C, diluted to 2 × 10^6^ CFUmL^−1^, and incubated with samples under test. Following an overnight incubation at 37 °C, each sample was diluted, plated on TSA, and incubated at 37 °C for 24 h, to evaluate the number of CFUmL^−1^. By using this experimental procedure, MBC values were determined as the lowest peptide concentration responsible for >99.9% of cell death. All the experiments were carried out in three independent replicates.

### 4.8. Kinetic Analysis of the Antimicrobial Activity of PDMS Polymer Loaded with r(P)ApoB_L_^Pro^ Peptide

In order to kinetically analyze the antibacterial effects of functionalized PDMS, the growth of *E. coli* ATCC 25922 cells was monitored over time (0–30–90–150–270–960 min) under three different experimental conditions, i.e., (i) bacteria alone, (ii) bacteria incubated with control PDMS, and (iii) bacteria incubated with PDMS functionalized with 10 µM r(P)ApoB_L_^Pro^. To this purpose, bacteria were grown to mid-logarithmic phase in MHB at 37 °C and then diluted to 4 × 10^6^ CFUmL^−1^ in 0.5X NB. Two hundred microliters of bacterial cells were then added to functionalized PDMS. At each time point, bacterial aliquots were withdrawn from the wells, serially diluted, and 100 μL of each dilution was plated on TSA in order to count bacterial colonies after an incubation of 16 h at 37 °C.

### 4.9. Evaluation of Peptide Release from PDMS by Quartz Crystal Microbalance (QCM) Analyses

To evaluate peptide release from PDMS, quartz oscillators (151218) from ICM (Oklahoma City, OK, USA) were used. Employed AT-CUT quartzes have a fundamental frequency of 10 MHz [57,58]. The crystal and the gold electrode diameters are 1.37 cm and 0.68 cm, respectively. To perform the analyses, the gold surfaces were cleaned by immersion of the oscillators for 1 min in a Piranha solution (concentrated sulfuric acid and 40% hydrogen peroxide 5:1 *v*/*v*). Afterwards, the quartzes were washed with pure water. The whole cleaning procedure was performed in the hood. The employed QCM device is a µLibra from Technobiochip, Italy [59]. To perform the analyses, the gold-quartz wafer was placed on the electronic console and the resonance frequency of the oscillator was monitored by using a proper software. To analyze the release of the peptide from the PDMS surface, oscillation frequency changes (Δf) of the piezoelectric quartz crystal were related to the amount of mass adsorbed or desorbed from the sensor surface detected in real time and with high sensitivity [60]. To do this, PDMS functionalized with 10 µM r(P)ApoB_L_^Pro^ was adsorbed on the gold sensor surface of the quartz microbalance and peptide release was monitored at defined time intervals by measuring the variation of the oscillation frequency upon addition and removal of a selected amount of medium. Specifically, 100 µL of 0.5X NB was added and removed at defined time intervals. Every time, data were recorded starting from 10 min after medium removal in order to allow the stabilization of oscillation frequency.

### 4.10. Evaluation of the Stability of PDMS Polymer Loaded with r(P)ApoB_L_^Pro^ Peptide over Time

To determine whether the antimicrobial activity of PDMS-r(P)ApoB_L_^Pro^ is stable over time, samples of PDMS both unfunctionalized and functionalized with 10 µM r(P)ApoB_L_^Pro^ peptide were separately poured into 96-well microtiter plates and stored at 4 °C for 120 days. At defined time intervals (4–7–14–30–120 days), the antimicrobial activity of stored samples was evaluated by adding 100 µL of *E. coli* ATCC 25922 cells (2 × 10^6^ CFUmL^−1^ in 0.5X NB) into each well and incubating samples at 37 °C for 16 h. Following incubation, bacterial samples were serially diluted (from 10 to 10,000 folds) and 100 µL of each dilution were plated on TSA in order to count bacterial colonies after an incubation of 16 h at 37 °C.

### 4.11. Evaluation of the Anti-Infective Properties of PDMS Polymer Loaded with r(P)ApoB_L_^Pro^ Peptide

To evaluate the anti-infective properties of PDMS functionalized with ApoB-derived peptide, PDMS alone and PDMS functionalized with increasing concentrations (10–20–30 µM) of r(P)ApoB_L_^Pro^ peptide were cut into slides of 1 cm^2^ each. Each slide was then placed into a 24-well microtiter plate and UV sterilized for 15 min. Samples were then tested for their bactericidal and anti-adhesive properties against *E. coli* ATCC 25922 bacterial cells. To this purpose, a single colony of the *E. coli* ATCC 25922 strain was transferred into 5 mL of MHB and grown at 37 °C to mid-log phase. Afterwards, 1 mL of bacterial suspension was diluted to 1 × 10^3^ CFUmL^−1^ in 0.5X NB and added to each sample to be incubated at 37 °C for 16 h. Following the incubation, the supernatant of bacterial culture was transferred from each well to a sterile Eppendorf tube, diluted, and plated for the evaluation of planktonic cells growth. Each slide was, instead, rinsed with 1 mL of sterile Hanks’ Balanced Salt Solution (HBSS), in order to remove the non-adherent bacteria, and then transferred into a sterile Eppendorf tube containing 1 mL of HBSS. In this case, adherent bacteria were detached from each slide by vortexing for 1 min, sonicating for 3 min in a water bath, vortexing again for 1 min, and centrifuging at 10,000 rpm for 5 min. Upon dilution, 100 μL of each sample were plated on TSA as duplicates, in order to count bacterial colonies after an incubation of 16 h at 37 °C.

### 4.12. Evaluation of the Antimicrobial Properties of PDMS Polymer Loaded with r(P)ApoB_L_^Pro^ Peptide by Scanning Electron Microscopy (SEM) Analyses

To perform SEM analyses, *E. coli* ATCC 25922 cells from an overnight culture were diluted to 1 × 10^9^ CFUmL^−1^ in 0.5 X MHB and placed into 24-well microtiter plates containing slides of PDMS alone or of PDMS functionalized with increasing concentrations (10–20–30 µM) of r(P)ApoB_L_^Pro^ for increasing lengths of time (16–24–72 h) at 37 °C in static conditions. At the end of the incubation, bacterial cells were processed and characterized as previously reported [27].

### 4.13. Evaluation of the Antibiofilm Properties of PDMS Polymer Loaded with r(P)ApoB_L_^Pro^ Peptide by Crystal Violet Assay

To test the antibiofilm activity of PDMS functionalized with r(P)ApoB_L_^Pro^ peptide, *E. coli* ATCC 25922 bacterial cells were grown overnight in MHB and diluted to 1 × 10^9^ CFUmL^−1^ in 0.5X MHB. PDMS alone and PDMS functionalized with increasing concentrations (10–20–30 µM) of r(P)ApoB_L_^Pro^ peptide were UV sterilized and placed into 24-well plates to be incubated with 0.5 mL of the bacterial suspension. At the end of the incubation carried out in static conditions at 37 °C for 24–72 h, crystal violet assays were performed as previously described [24]. At the end of the procedure, samples optical absorbance was determined at 630 nm by using a microtiter plate reader (FLUOstar Omega, BMG LABTECH, Ortenberg, Germany).

### 4.14. Analysis of the Biocompatibility of PDMS Polymer Loaded with r(P)ApoB_L_^Pro^ Peptide

To analyze the biocompatibility of PDMS samples, effects on the viability of murine BALB/c-3T3 fibroblasts and of human dermal fibroblasts (HDF) were evaluated. To this purpose, cells were cultured in Dulbecco’s Modified Eagle’s Medium (DMEM) supplemented with 10% fetal bovine serum (FBS), 1% antibiotics (pen/strep), and 1% L-glutamine at 37 °C in a humidified atmosphere containing 5% CO_2_. Cells, once confluent, were detached by using trypsin and 500 μL of cell suspension were seeded into a 24-well microtiter plate (2 × 10^4^ cells/well). Following an incubation overnight at 37 °C, PDMS slides (1 cm^2^) were added on the cells and samples were incubated at 37 °C for 24 h. Cytotoxicity was then evaluated by a 3-(4,5-dimethylthiazol-2-yl)-2,5 diphenyltetrazolium bromide (MTT) reduction inhibition assay. Medium and PDMS slides were removed and 300 μL of MTT reagent (0.5 mg/mL), dissolved in DMEM without phenol red, were added to the cells. The procedure was then carried out as previously described [61]. Cytotoxicity experiments were performed at least three times independently. Cell survival was expressed as the percentage of viable cells in contact with functionalized PDMS with respect to the sample incubated with unfunctionalized PDMS.

### 4.15. Statistical Analyses

Statistical analyses were performed using Student’s *t*-test. Significant differences were indicated as: * (*p* < 0.05), ** (*p* < 0.01), *** (*p* < 0.001) or **** (*p* < 0.0001).

## 5. Conclusions

An easy procedure to functionalize PDMS with r(P)ApoB_L_^Pro^ antimicrobial peptide has been here developed and the obtained functionalized material has been found to be stable, antimicrobial, and biocompatible. Indeed, this proof-of-concept study demonstrates for the first time the suitability of a natural ApoB-derived peptide to functionalize PDMS surface while retaining its antimicrobial properties. Further studies will be performed in the future by using a more resistant synthetic variant of the peptide, by testing its activity in a more physiological context and on clinically isolated bacterial strains, and by analyzing different immobilization procedures. Indeed, the obtained preliminary findings open interesting perspectives to the applicability of r(P)ApoB_L_^Pro^ peptide in the production of antimicrobial materials able to mitigate catheters associated bacterial infections.

## Figures and Tables

**Table 1:** Minimal bactericidal concentration (MBC) values of r(P)ApoB_L_^Pro^ and PDMS-r(P)ApoB_L_^Pro^.

	MBC (µM)	
Strains	r(P)ApoB_L_^Pro^	PDMS-r(P)ApoB_L_^Pro^
*Escherichia coli* ATCC 25922	5	10
*Staphylococcus aureus* ATCC 29213	10–20	80

## Data Availability

Data are contained within the article.
